# A combined chloroplast *atpB*–*rbcL* and *trnL*-*F* phylogeny unveils the ancestry of balsams (*Impatiens* spp.) in the Western Ghats of India

**DOI:** 10.1007/s13205-016-0574-8

**Published:** 2016-12-02

**Authors:** P. P. Shajitha, N. R. Dhanesh, P. J. Ebin, Joseph Laly, Devassy Aneesha, John Reshma, Jomy Augustine, Mathew Linu

**Affiliations:** 1 School of Biosciences, Mahatma Gandhi University, Kottayam, Kerala 686 560 India; 2 Department of Botany, St. Thomas College, Palai, Kottayam, Kerala 686 574 India; 3 Gurukula Botanical Sanctuary, Wayanad, Kerala 670 644 India

**Keywords:** *Impatiens* species, Molecular phylogeny, *atpB*–*rbcL* intergenic spacer, *trnL*-*F* spacer, Maximum parsimony, Bayesian inference

## Abstract

Only a few *Impatiens* spp. from South India (one of the five centers of diversity for *Impatiens* species) were included in the published datum of molecular phylogeny of the family *Balsaminaceae*. The present investigation is a novel attempt to reveal the phylogenetic association of *Impatiens* species of South India, by placing them in the global phylogeny of *Impatiens* based on a combined analysis of two chloroplast genes. Thirty species of genus *Impatiens* were collected from different locations of South India. Total genomic DNA was extracted from fresh plant leaf, and polymerase chain reaction was carried out using *atpB*–*rbcL* and *trnL*-*F* intergenic spacer-specific forward and reverse primers. Thirteen sequences of *Impatiens* species from three centers of diversity were obtained from GenBank for reconstructing the evolutionary relationships within the genus *Impatiens*. Bayesian inference analysis was carried out in MrBayes v.3.2.2. This analysis supported Southeast Asia as the ancestral place of origin of extant *Impatiens* species. Molecular phylogeny of South Indian *Impatiens* spp. based on combined chloroplast sequences showed the same association as that of morphological taxonomy. Sections *Scapigerae, Tomentosae, Sub*-*Umbellatae,* and *Racemosae* showed Southeast Asian relationship, while sections *Annuae* and *Microsepalae* showed African affinity.

**Electronic supplementary material:**

The online version of this article (doi:10.1007/s13205-016-0574-8) contains supplementary material, which is available to authorized users.

## Introduction

Two genera, namely, *Impatiens* and *Hydrocera,* are the sole members of the family *Balsaminaceae*. The genus *Hydrocera* is monotypic. *Impatiens* is a large genus containing more than 1000 species with a distribution pattern in the mountain areas of old world tropics and subtropics (Janssens et al. 2006). The five biodiversity hotspots for this highly endemic genus have been identified as Southeast Asia, Southern India and Sri Lanka, tropical Africa, Madagascar, and the Eastern Himalayas (Yuan et al. 2004; Janssens et al. 2006). Several novel species, belonging to this explosively speciating plant, are recognized in these regions every year (Kuang et al. 2014; Gogoi and Borah 2014; Luo et al. 2015). The genus *Impatiens* contains more than 210 species in India with amazing localization in two biodiversity hotspots, namely, Himalayas in the north of India and the Western Ghats in the south of India. Over half of these occur in the Western Ghats of India and at least 103 species of *Impatiens* are endemic to the Western Ghats alone (Bhaskar 2012).

Molecular phylogeny of *balsams* based on ITS sequences (Yuan et al. 2004) proposed that extant *Impatiens* species are of Southeast Asian origin, from where it dispersed to other parts of the globe in several dispersal events. Contrarily, *atpB*–*rbcL* intergenic spacer sequences based on phylogenetics of Janssens et al. (2006) suggested that *Impatiens* originated in South China from which it colonized the nearby regions and afterwards dispersed to north America, India, Africa, the Southeast Asian peninsula, and the Himalayan area. All these published data of molecular phylogeny and biogeography of *Balsaminaceae* inferred from ITS sequences (Yuan et al. 2004) and chloroplast *atpB*–*rbcL* spacer sequences (Janssens et al. 2006) contained only a few samples of *Impatiens* species from South India, creating a gap in the existing phylogeny of balsams. Hence, this work is a novel attempt on the molecular phylogeny of *Impatiens* species with representatives from six sections of balsams from South India.

## Materials and methods

Representative samples from the different sections of *Impatiens* species were collected from Southern Western Ghats of India. The plants were authenticated, and voucher specimens were deposited in the Herbarium of St. Thomas College (Palai, Kerala, India). The details of the sample collection were summarized in Table 1.

**Table 1:** Species used in this study with location, voucher no., and GenBank accession no. of *atpB*–*rbcL* and *trnL*-*F* sequences

SI. no.	Species name with section	Location^a^	Voucher no. of sample deposited	GenBank accession number	
				*atpB*–*rbcL*	*trnL*-*F*
Section: *Scapigerae*					
1	*I. levingei*	Eravikulam National Park	S.P.P.4854	KU316381	KU341090
2	*I. modesta*	Eravikulam National Park	S.P.P.4857	KU530217	KU341091
3	*I. pandata*	Eravikulam National Park	S.P.P.4856	KU316383	KU513967
4	*I. scapiflora*	Vagamon	S.P.P.4502	KF447374	KJ746922
Section: *Annuae*					
5	*I. aadishankarii*	Wayanad	S.P.P. 4546	KU316371	KU341086
6	*I. chinensis*	Munnar	S.P.P.4545	KU316374	KU341088
7	*I. dalzellii*	Eravikulam National Park	S.P.P.4852	KU316375	KU341089
8	*I. gardneriana*	Wayanad	S.P.P.4520	KF562062	KJ746912
9	*I. herbicola*	Neryamangalam	S.P.P.4505	KF562065	KJ746914
10	*I. ligulata*	Wayanad	S.P.P.4530	KF562063	KJ746916
11	*I. minor*	Neryamangalam	S.P.P.4504	KF447375	KJ703108
12	*I. oppositifolia*	Eravikulam National Park	S.P.P.4855	KU316382	KU341092
13	*I. raziana*	Eravikulam National Park	S.P.P.4851	KU316379	KU341093
14	*I. tomentosa*	Agasthyamala Biosphere Reserve	S.P.P.4861	KU316386	KU341094
Section: *Microcepalae*					
15	*I. bababudenensis*	Anamudi Hills	S.P.P.4548	KU316373	KU341087
16	*I. balsamina*	Munnar	S.P.P.4517	KF582043	KJ746906
17	*I. dasysperma*	Neryamangalam	S.P.P.4506	KM360163	KJ746909
18	*I. latifolia*	Eravikulam National Park	S.P.P.4549	KU316378	KU508414
19	*I. mysorensis*	Wayanad	S.P.P.4534	KF582048	KU508416
20	*I. pulcherrima*	Eravikulam National Park	S.P.P.4853	KU316384	KU508417
21	*I. scabriuscula*	Wayanad	S.P.P.4531	KF562058	KJ746921
22	*I. walleriana*	Munnar	S.P.P.4518	KF58205	0KJ746925
Section: *Tomentosae*					
23	*I. johnii*	Wayanad	S.P.P.4543	KU316377	KJ746915
24	*I. munronii*	Wayanad	S.P.P.4532	KF582047	KU508415
25	*I. neo*-*munronii*	Wayanad	S.P.P.4523	KF562061	KJ746919
Section: *Sub*-*Umbellatae*					
26	*I. cordata*	Munnar	S.P.P.4515	KF582044	KU508411
27	*I. disotis*	Wayanad	S.P.P.4528	KF582042	KU508412
28	*I. uncinata*	Wayanad	S.P.P.4529	KF562057	KJ746923
Section: *Racemosae*					
29	*I. maculata*	Devikulam	S.P.P.4507	KF562056	KJ746918
30	*I. wightiana*	Wayanad	S.P.P.4522	KF582052	KJ746926

^a^All locations in Kerala, India

Total genomic DNA was extracted using Gen Elute Plant Genomic DNA Miniprep Kit (Sigma Aldrich, St. Louis, USA). For PCR amplification, OrionX h-Taq PCR Smart Mix (Origin, India) was used. The primers used for the amplification of the chloroplast *atpB*–*rbcL* intergenic spacer gene were IMP-*atpB*—5′-ACATCTAGTACCGGACCAATGA-3′ and IMP-*rbcL*—5′-AACACCAGCTTTGAATCCAA-3′ (10 pM each) (Janssens et al. 2006), and *trnL*-*F* region were *trnL-F*—c:5′-CGAAATCGGTAGACGCTACG-3′ and *trnL-F*—f:5′-ATTTGAACTGGTGACACGAG-3′ (10 pM each) (Taberlet et al. 1991).

The temperature profile of amplification of *atpB*–*rbcL* intergenic spacer region was as per Janssens et al. (2006), and that of *trnL*-*F* region was as per Taberlet et al. (1991). Amplification reactions were carried out in an Agilent Sure Cycler 8800 (Agilent Technologies, USA) (ESM Figs. 1S, 2S). Amplicons (*atpB*–*rbcL* amplicon of size 900 bp and *trnL*-*F* amplicon of size 600–650 bp) were sequenced in AB1 cycle sequencer (Scigenome Labs Pvt. Ltd., Cochin, Kerala, India).

All sequences generated in this study were subjected to a BLAST search (NCBI) against the GenBank nucleotide database and submitted to GenBank (Table 1). *I. omeiana* was selected as outgroup for phylogenetic analyses of *Impatiens* (Janssens et al. 2009). Sequences of *Impatiens* species from three diversity hotspots were collected from GenBank accessions (Table 2). The sequences were multiple aligned and edited using the CLUSTALW (Thomson et al. 1994) program incorporated in BioEdit 7.0.5.2 (Hall 1999).

**Table 2:** Details of sequences of *atpB*–*rbcL* and *trnL*-*F* of *Impatiens* spp. obtained from GenBank

SI. no.	Place of origin and species name	Genbank accession number	
		*atpB*–*rbcL*	*trnL*-*F*
East and Southeast Asia			
1	*I. aquatilis*	DQ147811	KP776115
2	*I. davidi*	DQ147835	KP776129
3	*I. faberi*	DQ147841	KP776132
4	*I. gongshanensis*	KP776024	KP776135
5	*I. napoensis*	DQ147861	KP776146
6	*I. omeiana*	KC905619	KP776152
7	*I. platychlaena*	DQ147867	KP776154
8	*I. soulieana*	DQ147880	KP776164
9	*I. uliginosa*	DQ147887	KP776173
Africa			
10	*I. hians*	DQ147849	EF649977
11	*I. keilii*	FJ826654	KP776138
12	*I. mannii*	FJ826660	EF649980
Himalaya			
13	*I. scabrida*	DQ147877	KP776162

The Akaike information criterion (AIC) implemented in the program jModelTest version 2.1.5 (Darriba et al. 2012) was used to choose substitution models that best fit the data set. Bayesian inference analysis was carried out in MrBayes v.3.2.2 (Ronquist et al. 2012) in two independent runs, each with one heated chain and one cold chain and for one lakh generations. Convergence occurred when standard deviation (SD) of split frequencies fell below 0.05; the first 25% of MCMC generations were discarded as burn-in and a consensus phylogram was created. Posterior probability values were used to estimate branch support. Trees were visualized by Fig Tree, Tree Figure drawing tool version 1.4.2 (Rambaut 2014).

## Results and discussion

Phylogenetic analysis of this study included two chloroplast regions (*atpB*–*rbcL*, *trnL*-*F*) from 30 sequences of South Indian *Impatiens* species. In addition, 13 sequences of each of these regions were obtained from NCBI database. To assess the level of congruence between these data sets, each data set was analyzed independently to see if they produced a similar topology. The separate analyses produced topologies similar to each other. In comparison with separate analyses, the combined phylogeny had a well-resolved topology.

**Fig. 1:**
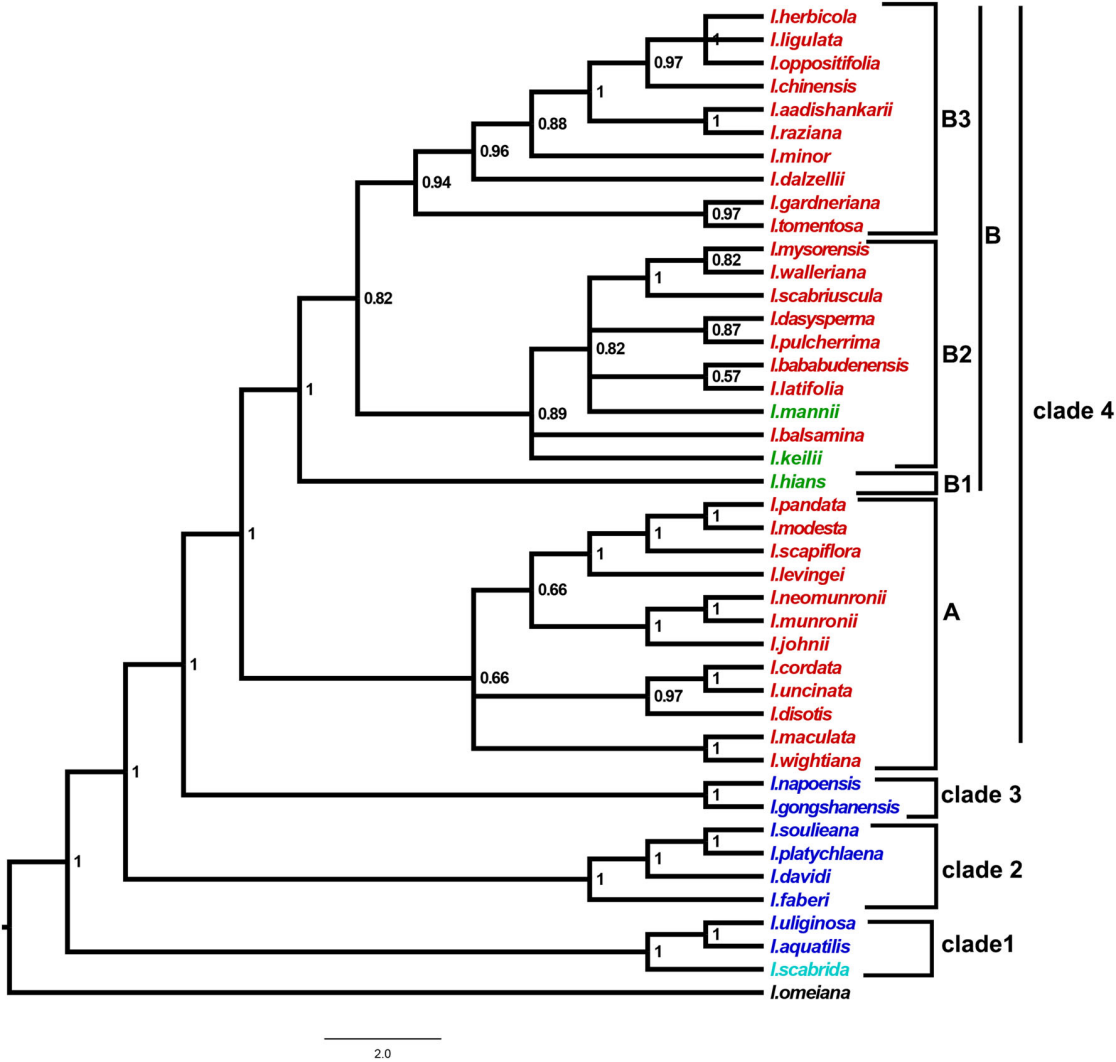
Bayesian consensus cladogram based on combined sequences of chloroplast *atpB*–*rbcL* intergenic spacer and *trnL*-*F* genes. The numbers by the nodes indicate Bayesian posterior probabilities greater than 0.5

The combined *atpB*–*rbcL* and *trnL*-*F* data matrix contained 1664 characters. A general time reversible model of evolution with invariant sites and a gamma distribution (GTR + I + G) was selected using jModelTest version 2.1.5. This model was used for the Bayesian inference (BI) analysis. The resulted tree by BI analysis had a well-resolved topology (Fig. 1). The resolved lineages of *Impatiens* species were grouped into four clades with strong Bayesian posterior probability (BPP) values. Two Southeast Asian species and the Himalayan species formed clade 1. Clade 2 included four Southeast Asian species. Two Southeast Asian species formed clade 3. Clade 4 was divided into two subclades, i.e., A and B (BPP of 1.00). Subclade A contained species of sections *Racemosae*, *Sub*-*Umbellatae, Tomentosae,* and *Scapigerae* (BPP of 1.00). Subclade B is divided into three subclades, i.e., B1, B2, and B3. African species (*I. hians*) formed Subclade B1. Subclade B2 included African species (*I. keilii* and *I. mannii*) and South Indian species of section *Microsepalae* with BPP of 0.89. Species of section *Annuae* produced Subclade B3 with BPP of 0.94.

## Implications on infrageneric classification and biogeography of *Impatiens* species of Western Ghats

*Impatiens* is considered taxonomically as one of the most difficult genera of angiosperms, mainly due to hypervariable structure and fragile nature of its flowers making examinations of dried specimen extremely difficult (Grey-Wilson 1980). The important revision of the African taxa by Grey-Wilson (1980) distinguished six informal infrageneric groups for the African species for practical diagnosis. Based on morphological and molecular data sets, Yu et al. (2015) presented a new classification of *Impatiens*, with the genus being divided into two subgenera, subgenus *Clavicarpa* and subgenus *Impatiens*. The subgenus *Impatiens* was further subdivided into seven sections.

In the taxonomic treatments of South Indian *Impatiens* by Bhaskar (2012), balsams of South India were classified under seven sections, i.e., *Scapigerae*, *Epiphyticae*, *Annuae*, *Microsepalae*, *Tomentosae*, *Sub-Umbellatae,* and *Racemosae*. Based on the present molecular phylogenetic study, species of each section formed monophyletic association with strong BPP support. This study authenticates the morphological classifications of Bhaskar (2012).

Based on several morphological similarities among species endemic to Africa and South India, close affinity between African and South Indian taxa and a possible migration route connecting these two areas were suggested (Grey-Wilson 1980). In this study, species of sections *Microsepalae* and *Annuae* showed African affinities with sister–clade relationships. This confirms Grey-Wilson’s (1980) suggestions of affinity between African and South Indian species. Sections *Scapigerae*, *Sub*-*Umbellatae*, *Tomentosae,* and *Racemosae* formed a separate clade (Subclade A) with sister–clade relationships with the extant Southeast Asian species.

There are several hypotheses related to the origin of *Impatiens* (Jones and Smith 1966; Grey-Wilson 1980). Bhaskar (1981) proposed that Western Ghats is the place of origin of the genus *Impatiens.* His hypothesis was based on the observation that Western Ghats of India contains the phylogenetically old species with primitive radial pollen grains, diploid chromosome number, and shrubby habit.

ITS phylogeny of Yuan et al. (2004) and *atpB*–*rbcL* phylogeny of Janssens et al. (2006) revealed that *Impatiens* spp. colonized African continent from Southwest China in three independent dispersal events. Madagascan species was derived from a single colonization event (Janssens et al. 2009). The present combined chloroplast gene analysis contained only three African and no Madagascan species. In this African species, *I. keilii* and *I. mannii* were placed with species of section *Microsepalae*. Section *Annuae* formed a sister–clade with this section. Himalayan species (*I. scabrida*) showed affinity to Southeast Asian species (*I. aquatilis* and *I. uliginosa*).

The biogeographical elucidation based on the present study is mainly in accordance with the conclusion of Yuan et al. (2004). The present analysis postulated that South India was colonized by two independent dispersal events, i.e., once by Southeast Asian ancestor as shown by the sister–clade relationships of extant Southeast Asian species and the sections *Scapigerae, Tomentosae, Sub*-*Umbellatae,* and *Racemosae* and a more recent colonization by an ancestor with African affinities (sections *Microsepalae* and *Annuae*).

## Electronic supplementary material

Below is the link to the electronic supplementary material.

Supplementary material 1 (TIFF 56 kb)

Supplementary material 2 (TIFF 50 kb)
