# Supplementary figures and images for: A combined chloroplast atpB–rbcL and trnL-F phylogeny unveils the ancestry of balsams (Impatiens spp.) in the Western Ghats of India
Source: 3 Biotech. 2016 Dec 2;6(2):258. doi: 10.1007/s13205-016-0574-8 (PMC5135705; doi:10.1007/s13205-016-0574-8)

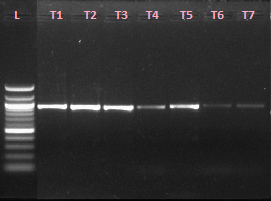

Supplement: Supplementary file 1 — Supplementary material 1 (TIFF 56 kb) [file 13205_2016_574_MOESM1_ESM.tif]

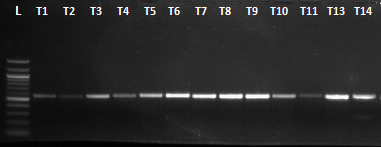

Supplement: Supplementary file 2 — Supplementary material 2 (TIFF 50 kb) [file 13205_2016_574_MOESM2_ESM.tif]
